# 2,4-Bis(morpholin-4-yl)-6-phen­oxy-1,3,5-triazine

**DOI:** 10.1107/S1600536811034088

**Published:** 2011-08-27

**Authors:** Jasmine P. Vennila, D. John Thiruvadigal, Helen P. Kavitha, G. Chakkaravarthi, V. Manivannan

**Affiliations:** a Department of Physics, Panimalar Institute of Technology, Chennai 602 103, India; b Department of Physics, SRM University, Kattankulathur Campus, Chennai, India; c Department of Chemistry, SRM University, Ramapuram Campus, Chennai 600 089, India; d Department of Physics, CPCL Polytechnic College, Chennai 600 068, India; e Department of Research and Development, PRIST University, Vallam, Thanjavur 613 403, Tamil Nadu, India

## Abstract

In the title compound, C_17_H_21_N_5_O_3_, the dihedral angle between the triazine and the phenyl ring is 80.31 (11)°. One of the morpholine rings is disordered over two orientations with site occupancies of 0.762 (10) and 0.238 (10). Both morpholine rings in the mol­ecule adopt chair conformations.

## Related literature

For triazine derivatives, see: Azev *et al.* (2003 ▶); Steffensen & Simanek (2003 ▶). For related structures, see: Zeng *et al.* (2005 ▶); Jian *et al.* (2007 ▶); Vennila *et al.* (2011 ▶). For puckering and asymmetry parameters, see: Cremer & Pople (1975 ▶). 
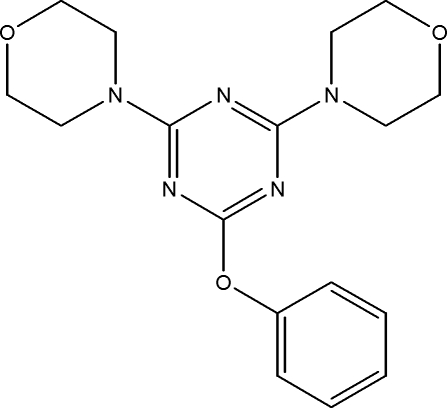


## Experimental

### 

#### Crystal data

C_17_H_21_N_5_O_3_
*M* *_r_* = 343.39
Orthorhombic, 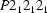

*a* = 8.6788 (5) Å
*b* = 11.2461 (5) Å
*c* = 17.9778 (10) Å
*V* = 1754.68 (16) Å^3^
*Z* = 4
Mo *K*α radiation
μ = 0.09 mm^−1^
*T* = 295 K
0.30 × 0.24 × 0.20 mm

#### Data collection

Bruker Kappa APEXII diffractometer
Absorption correction: multi-scan (*SADABS*; Sheldrick, 1996 ▶) *T*_min_ = 0.973, *T*_max_ = 0.982
18679 measured reflections
2028 independent reflections
1613 reflections with *I* > 2σ(*I*)
*R*_int_ = 0.031

#### Refinement

*R*[*F* ^2^ > 2σ(*F* ^2^)] = 0.041
*wR*(*F* ^2^) = 0.115
*S* = 1.05
2028 reflections
272 parameters
H-atom parameters constrained
Δρ_max_ = 0.16 e Å^−3^
Δρ_min_ = −0.14 e Å^−3^

### 

Data collection: *APEX2* (Bruker, 2004 ▶); cell refinement: *SAINT* (Bruker, 2004 ▶); data reduction: *SAINT*; program(s) used to solve structure: *SHELXS97* (Sheldrick, 2008 ▶); program(s) used to refine structure: *SHELXL97* (Sheldrick, 2008 ▶); molecular graphics: *PLATON* (Spek, 2009 ▶); software used to prepare material for publication: *SHELXL97*.

## Supplementary Material

Crystal structure: contains datablock(s) global, I. DOI: 10.1107/S1600536811034088/bt5604sup1.cif

Structure factors: contains datablock(s) I. DOI: 10.1107/S1600536811034088/bt5604Isup2.hkl

Supplementary material file. DOI: 10.1107/S1600536811034088/bt5604Isup3.cml

Additional supplementary materials:  crystallographic information; 3D view; checkCIF report

## Figures and Tables

**Fig. 1.**
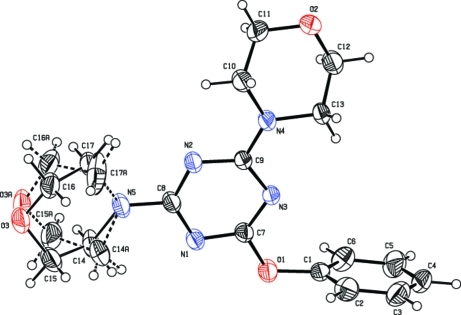
The molecular structure of (I), with atom labels and 30% probability displacement ellipsoids for non-H atoms.

## supplementary crystallographic information

### Comment

1,3,5-Triazine derivatives are of importance   as starting materials for drugs and light stabilizers (Azev *et al.*, 2003; Steffensen & Simanek, 2003). The bond lengths and angles are comparable with similar reported structures (Zeng *et al.*, 2005; Jian *et al.*, 2007; Vennila *et al.*, 2011). The dihedral angle between the triazine (C7/N1/C8/N2/C9/N3) ring and the phenyl ring (C1—C6) is 80.31 (11)°.

In the molecule, one of the morpholine rings is disordered over two orientations with site occupancies of 0.762 (10) and 0.238 (10). The morpholine ring N4/C10/C11/O2/C12/C13 and the disordered morpholine ring [with major site occupancy orientation (N5/C14/C15/O3/C16/C17) and minor site occupancy orientation (N5A/C14A/C15A/O3A/C16A/C17A)] adopt chair conformations (Cremer & Pople, 1975): Q = 0.547 (3) Å, θ = 175.9 (3)°, Φ = 174 (5)° for the ring N4/C10/C11/O2/C12/C13; Q = 0.551 (6) Å, θ = 3.1 (7)°, Φ = 246 (11)° for the disordered ring (N5/C14/C15/O3/C16/C17) and Q = 0.68 (2) Å, θ = 167.6 (15)°, Φ = 169 (9)° for the disordered ring (N5A/C14A/C15A/O3A/C16A/C17A).

### Experimental

A solution of cyanuric chloride (1.85 g, 10 mmol) in 6 ml acetone was added with stirring to a cold solution (0–5°C) of sodium bicarbonate (0.85 g, 10 mmol) in 10 ml of distilled water in a three necked flask equipped with a mechanical stirrer. This resulted in the formation of a slurry of cyanuric chloride. A solution of phenol (1 ml) in 5 ml acetone was added to the cold slurry of cyanuric chloride. After some time, the reaction mixture was neutralized by a saturated solution of sodium bicarbonate. The mixture was stirred for 2 h at 0–5°C. The crude product obtained was filtered and recrystallized from ethanol. To this product (1.75 g, 5 mmol) in acetone (5 ml), a mixture of NaOH (0.08 g, 20 mmol) and morpholine (1 ml) in 8 ml double distilled water was added slowly at room temperature with constant stirring. Stirring was continued for 4 h initially for 2 h at room temperature and another 2 h at 80°C. The crude product was filtered and recystallized from ethanol to yield colourless diffraction quality crystals.

### Refinement

Due to the absence of anomalous scatterers, 1495 Friedel pairs have been merged. Site occupancy factors of disordered atoms in the morpholine ring (A) were refined as 0.762 (10) for major orientation (N5/C14/C15/O3/C16/C17) and 0.238 (10) for minor orientation (N5A/C14A/C15A/O3A/C16A/C17A). All H atoms were positioned geometrically with C—H = 0.93–0.97 Å and allowed to ride on their parent atoms, with *U*_iso_(H) = 1.2Ueq(C).

### Crystal data

**Table:**

C_17_H_21_N_5_O_3_	*F*(000) = 728
*M**_r_* = 343.39	*D*_x_ = 1.300 Mg m^−^^3^
Orthorhombic, *P*2_1_2_1_2_1_	Mo *K*α radiation, λ = 0.71073 Å
Hall symbol: P 2ac 2ab	Cell parameters from 5915 reflections
*a* = 8.6788 (5) Å	θ = 2.3–26.2°
*b* = 11.2461 (5) Å	µ = 0.09 mm^−^^1^
*c* = 17.9778 (10) Å	*T* = 295 K
*V* = 1754.68 (16) Å^3^	Block, colourless
*Z* = 4	0.30 × 0.24 × 0.20 mm

### Data collection

**Table:**

Bruker Kappa APEXII diffractometer	2028 independent reflections
Radiation source: fine-focus sealed tube	1613 reflections with *I* > 2σ(*I*)
graphite	*R*_int_ = 0.031
ω and φ scans	θ_max_ = 26.3°, θ_min_ = 2.1°
Absorption correction: multi-scan (*SADABS*; Sheldrick, 1996)	*h* = −10→10
*T*_min_ = 0.973, *T*_max_ = 0.982	*k* = −12→14
18679 measured reflections	*l* = −22→22

### Refinement

**Table:**

Refinement on *F*^2^	Primary atom site location: structure-invariant direct methods
Least-squares matrix: full	Secondary atom site location: difference Fourier map
*R*[*F*^2^ > 2σ(*F*^2^)] = 0.041	Hydrogen site location: inferred from neighbouring sites
*wR*(*F*^2^) = 0.115	H-atom parameters constrained
*S* = 1.05	*w* = 1/[σ^2^(*F*_o_^2^) + (0.0642*P*)^2^ + 0.2172*P*] where *P* = (*F*_o_^2^ + 2*F*_c_^2^)/3
2028 reflections	(Δ/σ)_max_ < 0.001
272 parameters	Δρ_max_ = 0.16 e Å^−^^3^
0 restraints	Δρ_min_ = −0.14 e Å^−^^3^

### Fractional atomic coordinates and isotropic or equivalent isotropic displacement parameters (Å^2^)

**Table:**

	*x*	*y*	*z*	*U*_iso_*/*U*_eq_	Occ. (<1)
O1	0.2358 (3)	0.06378 (14)	0.28176 (9)	0.0714 (6)	
O2	0.2262 (3)	0.28572 (15)	0.65046 (9)	0.0675 (6)	
N1	0.2683 (3)	−0.08734 (16)	0.35905 (11)	0.0546 (6)	
N2	0.3107 (3)	−0.05145 (16)	0.48804 (11)	0.0585 (6)	
N3	0.2685 (3)	0.11092 (15)	0.40465 (9)	0.0504 (5)	
N4	0.3066 (4)	0.14047 (16)	0.52974 (11)	0.0696 (8)	
N5	0.3017 (4)	−0.24105 (17)	0.44123 (12)	0.0736 (8)	
C1	0.2359 (4)	0.1847 (2)	0.26391 (12)	0.0534 (7)	
C2	0.3713 (4)	0.2430 (3)	0.25415 (18)	0.0691 (8)	
H2	0.4647	0.2052	0.2634	0.083*	
C3	0.3672 (5)	0.3596 (3)	0.23017 (18)	0.0786 (10)	
H3	0.4591	0.4006	0.2229	0.094*	
C4	0.2318 (5)	0.4150 (3)	0.21713 (15)	0.0771 (10)	
H4	0.2307	0.4939	0.2017	0.092*	
C5	0.0974 (5)	0.3553 (3)	0.22666 (19)	0.0813 (10)	
H5	0.0043	0.3934	0.2171	0.098*	
C6	0.0975 (4)	0.2388 (3)	0.25034 (18)	0.0687 (8)	
H6	0.0055	0.1978	0.2570	0.082*	
C7	0.2598 (3)	0.02907 (19)	0.35318 (12)	0.0487 (6)	
C8	0.2933 (3)	−0.1225 (2)	0.42922 (13)	0.0543 (6)	
C9	0.2951 (3)	0.06457 (19)	0.47306 (12)	0.0513 (6)	
C10	0.3399 (5)	0.1037 (2)	0.60578 (14)	0.0746 (10)	
H10A	0.4439	0.1271	0.6191	0.089*	
H10B	0.3325	0.0178	0.6098	0.089*	
C11	0.2281 (5)	0.1604 (2)	0.65710 (14)	0.0734 (9)	
H11A	0.1258	0.1298	0.6468	0.088*	
H11B	0.2542	0.1392	0.7079	0.088*	
C12	0.1844 (4)	0.3172 (2)	0.57681 (16)	0.0715 (9)	
H12A	0.1807	0.4031	0.5726	0.086*	
H12B	0.0822	0.2866	0.5662	0.086*	
C13	0.2946 (5)	0.2695 (2)	0.52140 (14)	0.0703 (9)	
H13A	0.2596	0.2888	0.4716	0.084*	
H13B	0.3950	0.3057	0.5287	0.084*	
C14	0.2687 (13)	−0.3314 (8)	0.3851 (5)	0.078 (3)	0.701 (10)
H14A	0.1642	−0.3603	0.3914	0.093*	0.701 (10)
H14B	0.2774	−0.2968	0.3359	0.093*	0.701 (10)
C15	0.3741 (12)	−0.4272 (4)	0.3923 (3)	0.099 (3)	0.701 (10)
H15A	0.3464	−0.4884	0.3567	0.119*	0.701 (10)
H15B	0.4764	−0.3991	0.3795	0.119*	0.701 (10)
O3	0.3791 (8)	−0.4783 (4)	0.4637 (3)	0.0897 (16)	0.701 (10)
C16	0.4171 (8)	−0.3914 (4)	0.5164 (3)	0.0720 (15)	0.701 (10)
H16A	0.5182	−0.3592	0.5052	0.086*	0.701 (10)
H16B	0.4214	−0.4274	0.5654	0.086*	0.701 (10)
C17	0.3037 (12)	−0.2952 (7)	0.5166 (4)	0.071 (2)	0.701 (10)
H17A	0.3314	−0.2358	0.5534	0.086*	0.701 (10)
H17B	0.2026	−0.3261	0.5288	0.086*	0.701 (10)
C14A	0.305 (5)	−0.314 (2)	0.3745 (16)	0.158 (16)	0.299 (10)
H14C	0.4099	−0.3356	0.3611	0.189*	0.299 (10)
H14D	0.2568	−0.2740	0.3328	0.189*	0.299 (10)
C15A	0.214 (3)	−0.4207 (11)	0.3988 (7)	0.104 (7)	0.299 (10)
H15C	0.1068	−0.3977	0.4042	0.124*	0.299 (10)
H15D	0.2192	−0.4807	0.3601	0.124*	0.299 (10)
O3A	0.265 (3)	−0.4716 (10)	0.4665 (7)	0.123 (7)	0.299 (10)
C16A	0.273 (5)	−0.3884 (12)	0.5234 (7)	0.147 (13)	0.299 (10)
H16C	0.3127	−0.4283	0.5672	0.177*	0.299 (10)
H16D	0.1688	−0.3632	0.5348	0.177*	0.299 (10)
C17A	0.368 (4)	−0.279 (2)	0.5111 (16)	0.128 (13)	0.299 (10)
H17C	0.3526	−0.2209	0.5502	0.154*	0.299 (10)
H17D	0.4765	−0.2975	0.5066	0.154*	0.299 (10)

### Atomic displacement parameters (Å^2^)

**Table:**

	*U*^11^	*U*^22^	*U*^33^	*U*^12^	*U*^13^	*U*^23^
O1	0.1290 (18)	0.0440 (8)	0.0412 (9)	0.0023 (12)	−0.0188 (11)	−0.0017 (7)
O2	0.0994 (15)	0.0550 (10)	0.0481 (9)	0.0018 (11)	0.0051 (10)	−0.0090 (8)
N1	0.0788 (16)	0.0392 (9)	0.0457 (10)	0.0056 (10)	−0.0109 (11)	−0.0036 (8)
N2	0.0933 (18)	0.0368 (9)	0.0456 (11)	0.0045 (11)	−0.0062 (12)	−0.0008 (8)
N3	0.0768 (15)	0.0358 (8)	0.0387 (9)	0.0018 (11)	−0.0034 (10)	−0.0004 (7)
N4	0.132 (2)	0.0381 (10)	0.0388 (11)	0.0057 (13)	−0.0024 (13)	−0.0010 (8)
N5	0.132 (2)	0.0355 (10)	0.0535 (12)	0.0106 (13)	−0.0188 (15)	−0.0033 (9)
C1	0.082 (2)	0.0453 (11)	0.0325 (10)	0.0048 (14)	−0.0042 (12)	−0.0001 (9)
C2	0.069 (2)	0.073 (2)	0.0656 (18)	0.0070 (16)	−0.0059 (15)	0.0014 (16)
C3	0.091 (2)	0.077 (2)	0.068 (2)	−0.020 (2)	0.0049 (17)	0.0118 (17)
C4	0.124 (3)	0.0561 (15)	0.0506 (14)	0.003 (2)	−0.0017 (19)	0.0153 (12)
C5	0.087 (2)	0.074 (2)	0.083 (2)	0.0228 (19)	−0.0038 (17)	0.0235 (18)
C6	0.0678 (19)	0.073 (2)	0.0654 (18)	−0.0020 (16)	−0.0034 (14)	0.0142 (16)
C7	0.0647 (16)	0.0413 (11)	0.0402 (11)	0.0033 (12)	−0.0062 (11)	−0.0020 (9)
C8	0.0754 (18)	0.0386 (11)	0.0490 (13)	0.0083 (13)	−0.0081 (13)	−0.0040 (10)
C9	0.0766 (17)	0.0369 (10)	0.0403 (11)	0.0040 (12)	0.0015 (12)	−0.0008 (9)
C10	0.130 (3)	0.0500 (13)	0.0435 (13)	0.0165 (18)	−0.0122 (17)	−0.0005 (11)
C11	0.117 (3)	0.0575 (15)	0.0458 (13)	−0.0086 (18)	0.0039 (16)	0.0026 (11)
C12	0.102 (2)	0.0504 (13)	0.0623 (16)	0.0093 (16)	−0.0080 (16)	−0.0036 (13)
C13	0.125 (3)	0.0349 (11)	0.0510 (14)	0.0004 (15)	0.0050 (17)	−0.0031 (10)
C14	0.148 (6)	0.032 (3)	0.054 (3)	−0.004 (3)	−0.041 (4)	0.001 (2)
C15	0.186 (8)	0.050 (3)	0.062 (3)	0.038 (4)	−0.004 (4)	−0.005 (2)
O3	0.159 (5)	0.0407 (18)	0.070 (2)	0.024 (3)	−0.001 (3)	0.0062 (15)
C16	0.102 (4)	0.048 (2)	0.066 (3)	0.003 (3)	−0.011 (3)	0.011 (2)
C17	0.127 (7)	0.036 (3)	0.051 (3)	−0.001 (4)	−0.011 (4)	0.000 (2)
C14A	0.33 (4)	0.042 (9)	0.106 (15)	−0.014 (15)	0.073 (19)	−0.017 (9)
C15A	0.18 (2)	0.054 (7)	0.077 (8)	−0.033 (10)	0.003 (10)	−0.002 (6)
O3A	0.26 (2)	0.037 (4)	0.076 (6)	−0.022 (9)	−0.014 (12)	0.005 (4)
C16A	0.34 (4)	0.059 (8)	0.046 (6)	−0.057 (16)	0.001 (13)	0.005 (5)
C17A	0.19 (3)	0.055 (9)	0.135 (18)	−0.042 (13)	−0.100 (19)	0.032 (9)

### Geometric parameters (Å, °)

**Table:**

O1—C7	1.358 (3)	C11—H11A	0.9700
O1—C1	1.397 (3)	C11—H11B	0.9700
O2—C11	1.415 (3)	C12—C13	1.481 (4)
O2—C12	1.418 (3)	C12—H12A	0.9700
N1—C7	1.315 (3)	C12—H12B	0.9700
N1—C8	1.340 (3)	C13—H13A	0.9700
N2—C8	1.334 (3)	C13—H13B	0.9700
N2—C9	1.339 (3)	C14—C15	1.420 (11)
N3—C7	1.307 (3)	C14—H14A	0.9700
N3—C9	1.356 (3)	C14—H14B	0.9700
N4—C9	1.333 (3)	C15—O3	1.407 (7)
N4—C10	1.457 (3)	C15—H15A	0.9700
N4—C13	1.463 (3)	C15—H15B	0.9700
N5—C8	1.352 (3)	O3—C16	1.401 (7)
N5—C17A	1.45 (3)	C16—C17	1.463 (11)
N5—C14A	1.45 (3)	C16—H16A	0.9700
N5—C14	1.460 (9)	C16—H16B	0.9700
N5—C17	1.485 (9)	C17—H17A	0.9700
C1—C2	1.357 (4)	C17—H17B	0.9700
C1—C6	1.368 (4)	C14A—C15A	1.50 (3)
C2—C3	1.381 (4)	C14A—H14C	0.9700
C2—H2	0.9300	C14A—H14D	0.9700
C3—C4	1.351 (5)	C15A—O3A	1.415 (19)
C3—H3	0.9300	C15A—H15C	0.9700
C4—C5	1.357 (5)	C15A—H15D	0.9700
C4—H4	0.9300	O3A—C16A	1.389 (17)
C5—C6	1.378 (4)	C16A—C17A	1.49 (3)
C5—H5	0.9300	C16A—H16C	0.9700
C6—H6	0.9300	C16A—H16D	0.9700
C10—C11	1.483 (4)	C17A—H17C	0.9700
C10—H10A	0.9700	C17A—H17D	0.9700
C10—H10B	0.9700		
			
C7—O1—C1	119.79 (17)	C13—C12—H12B	109.2
C11—O2—C12	109.3 (2)	H12A—C12—H12B	107.9
C7—N1—C8	112.24 (19)	N4—C13—C12	109.6 (2)
C8—N2—C9	114.4 (2)	N4—C13—H13A	109.7
C7—N3—C9	112.42 (18)	C12—C13—H13A	109.7
C9—N4—C10	123.4 (2)	N4—C13—H13B	109.7
C9—N4—C13	123.5 (2)	C12—C13—H13B	109.7
C10—N4—C13	113.1 (2)	H13A—C13—H13B	108.2
C8—N5—C17A	116.9 (10)	C15—C14—N5	109.9 (5)
C8—N5—C14A	115.2 (12)	C15—C14—H14A	109.7
C17A—N5—C14A	122.7 (18)	N5—C14—H14A	109.7
C8—N5—C14	124.4 (4)	C15—C14—H14B	109.7
C17A—N5—C14	118.1 (11)	N5—C14—H14B	109.7
C8—N5—C17	123.4 (3)	H14A—C14—H14B	108.2
C14A—N5—C17	121.4 (12)	O3—C15—C14	114.3 (6)
C14—N5—C17	110.3 (5)	O3—C15—H15A	108.7
C2—C1—C6	121.5 (2)	C14—C15—H15A	108.7
C2—C1—O1	120.0 (3)	O3—C15—H15B	108.7
C6—C1—O1	118.3 (3)	C14—C15—H15B	108.7
C1—C2—C3	118.5 (3)	H15A—C15—H15B	107.6
C1—C2—H2	120.7	C16—O3—C15	109.9 (4)
C3—C2—H2	120.7	O3—C16—C17	111.0 (5)
C4—C3—C2	121.0 (3)	O3—C16—H16A	109.4
C4—C3—H3	119.5	C17—C16—H16A	109.4
C2—C3—H3	119.5	O3—C16—H16B	109.4
C3—C4—C5	119.8 (3)	C17—C16—H16B	109.4
C3—C4—H4	120.1	H16A—C16—H16B	108.0
C5—C4—H4	120.1	C16—C17—N5	108.0 (6)
C4—C5—C6	120.6 (3)	C16—C17—H17A	110.1
C4—C5—H5	119.7	N5—C17—H17A	110.1
C6—C5—H5	119.7	C16—C17—H17B	110.1
C1—C6—C5	118.6 (3)	N5—C17—H17B	110.1
C1—C6—H6	120.7	H17A—C17—H17B	108.4
C5—C6—H6	120.7	N5—C14A—C15A	102 (2)
N3—C7—N1	129.8 (2)	N5—C14A—H14C	111.5
N3—C7—O1	118.40 (19)	C15A—C14A—H14C	111.5
N1—C7—O1	111.74 (18)	N5—C14A—H14D	111.5
N2—C8—N1	126.0 (2)	C15A—C14A—H14D	111.5
N2—C8—N5	117.2 (2)	H14C—C14A—H14D	109.3
N1—C8—N5	116.8 (2)	O3A—C15A—C14A	114.1 (18)
N4—C9—N2	117.6 (2)	O3A—C15A—H15C	108.7
N4—C9—N3	117.40 (19)	C14A—C15A—H15C	108.7
N2—C9—N3	125.0 (2)	O3A—C15A—H15D	108.7
N4—C10—C11	109.4 (3)	C14A—C15A—H15D	108.7
N4—C10—H10A	109.8	H15C—C15A—H15D	107.6
C11—C10—H10A	109.8	C16A—O3A—C15A	112.2 (12)
N4—C10—H10B	109.8	O3A—C16A—C17A	118.2 (19)
C11—C10—H10B	109.8	O3A—C16A—H16C	107.7
H10A—C10—H10B	108.2	C17A—C16A—H16C	107.7
O2—C11—C10	112.6 (3)	O3A—C16A—H16D	107.7
O2—C11—H11A	109.1	C17A—C16A—H16D	107.7
C10—C11—H11A	109.1	H16C—C16A—H16D	107.1
O2—C11—H11B	109.1	N5—C17A—C16A	98.9 (19)
C10—C11—H11B	109.1	N5—C17A—H17C	112.0
H11A—C11—H11B	107.8	C16A—C17A—H17C	112.0
O2—C12—C13	111.9 (3)	N5—C17A—H17D	112.0
O2—C12—H12A	109.2	C16A—C17A—H17D	112.0
C13—C12—H12A	109.2	H17C—C17A—H17D	109.7
O2—C12—H12B	109.2		
			
C7—O1—C1—C2	80.8 (3)	C7—N3—C9—N4	179.8 (3)
C7—O1—C1—C6	−104.8 (3)	C7—N3—C9—N2	−0.2 (4)
C6—C1—C2—C3	0.2 (4)	C9—N4—C10—C11	−131.2 (3)
O1—C1—C2—C3	174.4 (2)	C13—N4—C10—C11	51.6 (4)
C1—C2—C3—C4	0.4 (5)	C12—O2—C11—C10	60.2 (4)
C2—C3—C4—C5	−0.9 (5)	N4—C10—C11—O2	−55.6 (4)
C3—C4—C5—C6	0.7 (5)	C11—O2—C12—C13	−60.3 (4)
C2—C1—C6—C5	−0.4 (4)	C9—N4—C13—C12	130.6 (3)
O1—C1—C6—C5	−174.7 (3)	C10—N4—C13—C12	−52.2 (4)
C4—C5—C6—C1	−0.1 (5)	O2—C12—C13—N4	56.2 (4)
C9—N3—C7—N1	2.3 (4)	C8—N5—C14—C15	141.3 (6)
C9—N3—C7—O1	−179.0 (3)	C17A—N5—C14—C15	−30 (2)
C8—N1—C7—N3	−1.8 (5)	C14A—N5—C14—C15	81 (6)
C8—N1—C7—O1	179.4 (2)	C17—N5—C14—C15	−53.9 (11)
C1—O1—C7—N3	6.1 (4)	N5—C14—C15—O3	54.9 (11)
C1—O1—C7—N1	−174.9 (3)	C14—C15—O3—C16	−57.8 (9)
C9—N2—C8—N1	2.6 (4)	C15—O3—C16—C17	59.7 (8)
C9—N2—C8—N5	−177.5 (3)	O3—C16—C17—N5	−59.9 (8)
C7—N1—C8—N2	−0.9 (4)	C8—N5—C17—C16	−138.5 (5)
C7—N1—C8—N5	179.1 (3)	C17A—N5—C17—C16	−58 (3)
C17A—N5—C8—N2	−15.7 (17)	C14A—N5—C17—C16	43 (2)
C14A—N5—C8—N2	−171.2 (19)	C14—N5—C17—C16	56.5 (9)
C14—N5—C8—N2	173.0 (7)	C8—N5—C14A—C15A	−144.3 (15)
C17—N5—C8—N2	10.1 (7)	C17A—N5—C14A—C15A	62 (3)
C17A—N5—C8—N1	164.2 (16)	C14—N5—C14A—C15A	−17 (4)
C14A—N5—C8—N1	8.8 (19)	C17—N5—C14A—C15A	34 (3)
C14—N5—C8—N1	−7.1 (7)	N5—C14A—C15A—O3A	−52 (3)
C17—N5—C8—N1	−169.9 (5)	C14A—C15A—O3A—C16A	53 (3)
C10—N4—C9—N2	2.0 (5)	C15A—O3A—C16A—C17A	−55 (3)
C13—N4—C9—N2	178.9 (3)	C8—N5—C17A—C16A	146.4 (17)
C10—N4—C9—N3	−177.9 (3)	C14A—N5—C17A—C16A	−60 (4)
C13—N4—C9—N3	−1.0 (5)	C14—N5—C17A—C16A	−42 (3)
C8—N2—C9—N4	178.1 (3)	C17—N5—C17A—C16A	34 (2)
C8—N2—C9—N3	−1.9 (4)	O3A—C16A—C17A—N5	52 (3)

### Hydrogen-bond geometry (Å, °)

**Table:**

*D*—H···*A*	*D*—H	H···*A*	*D*···*A*	*D*—H···*A*
C10—H10B···N2	0.97	2.33	2.755 (3)	106
C13—H13A···N3	0.97	2.34	2.764 (3)	106
C14—H14B···N1	0.97	2.39	2.784 (9)	104
C17—H17A···N2	0.97	2.39	2.789 (9)	104
